# The Influence of Dietary Supplements on Exercise-Induced Gut Damage and Gastrointestinal Symptoms: A Systematic Review and Meta-Analysis

**DOI:** 10.3390/nu17030443

**Published:** 2025-01-25

**Authors:** Robyn Aitkenhead, Mark Waldron, Gillian E. Conway, Katy Horner, Shane M. Heffernan

**Affiliations:** 1 A-STEM Centre, Faculty of Science and Engineering, Swansea University, Swansea SA1 8EN, UK; robyn.aitkenhead@swansea.ac.uk (R.A.); s.m.heffernan@swansea.ac.uk (S.M.H.); 2 Welsh Institute of Performance Science, Swansea University, Swansea SA1 8EN, UK; 3 School of Health and Behavioral Sciences, University of the Sunshine Coast, Sippy Downs, QLD 4556, Australia; 4 In Vitro Toxicology Group, Institute of Life Science, Swansea University Medical School, Swansea SA2 8PP, UK; gillian.conway@swansea.ac.uk; 5 School of Public Health, Physiotherapy and Sport Science, University College, Belfield, D04 V1W8 Dublin, Ireland; katy.horner@ucd.ie

**Keywords:** gut damage, exercise, supplements, heat

## Abstract

Endurance exercise, especially under heat stress, temporarily compromises the integrity of the intestinal barrier in healthy individuals. Consequently, there is growing interest in developing effective dietary strategies to alleviate exercise-induced gastrointestinal symptoms and gut damage. This meta-analysis investigated the effects of dietary supplements on mitigating these challenges. The search was performed in November 2024 following PRISMA guidelines, and 26 peer-reviewed studies were included across three meta-analyses: (1) gastrointestinal symptoms, (2) circulating intestinal fatty acid-binding protein (i-FABP), and (3) exercise performance. The moderating effect of variables was assessed via sub-group analysis and meta-regression. Overall, there was no pooled effect of supplement interventions on gastrointestinal symptoms (Hedges’ g = 0.42, 95% CI −0.17: 1.02, *p* = 0.15), and probiotics had a moderate significant effect for gastrointestinal symptoms (Hedges’ g = −0.62, 95% CI −1.01; 1.01, *p* = 0.05). There was a significant increase in i-FABP concentrations pre- to post exercise (∆ 106%; Hedges’ g = 1.01, 95% CI 0.63; 1.38, *p* = 0.01). There were no pooled or sub-group differences for exercise performance for any supplements (*p* = 0.53). Moderate-to-large heterogeneity was observed across studies (I^2^ ≥ 58.6%), and candidate moderators (exercise duration, modality, and environmental temperature) had no significant effect on any outcomes (*p* > 0.05). A significant increase in circulating i-FABP during exercise was observed. However, when examining the effects of different supplement categories, although significance was observed for a select few supplements, the changes in i-FABP, gastrointestinal symptoms, and exercise performance were outside of clinical relevance. Although probiotics showed a moderate significant effect for gastrointestinal symptoms, the conflicting findings across studies may have been due to inadequate control of confounding variables across studies. Further research is required to assess the alternative dietary supplements’ effects on gastrointestinal health and exercise performance, particularly under varied environmental conditions, where more rigorous control for cofounding factors is implemented.

## 1. Introduction

**Figure 1:**
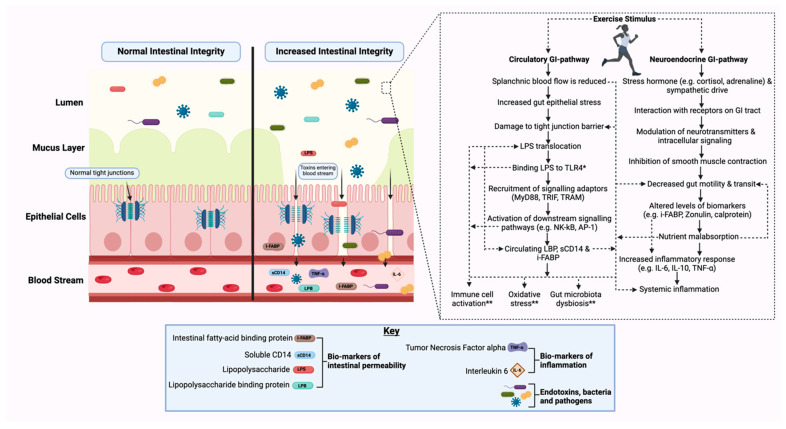
Normal intestinal integrity versus compromised intestinal integrity and the proposed signalling cascades in circulatory–gastrointestinal and neuroendocrine–gastrointestinal pathways. * and ** denotes hypothesised mechanism and response. Gastrointestinal, GI; lipopolysaccharides, LPSs; toll-like receptor 4, TLR4; myeloid differentiation factor 88, MyD88; TIR domain-containing adapter-inducing interferon-β, TRIF; TRIF-related adaptor molecule, TRAM; nuclear factor kappa B, NF-kB; activator protein 1, AP-1; lipopolysaccharide-binding protein, LBP; soluble cluster of differentiation 14, sCD14; intestinal fatty acid-binding protein, i-FABP; interleukin-6, IL-6; interleukin-10, IL-10; and tumour necrosis factor-alpha, TNF-.

The intestinal epithelium, particularly in the small intestine, lines the gastrointestinal (GI) tract, serving as the primary barrier against exogenous molecules [1,2]. This selectively permeable barrier allows for the absorption of essential nutrients and electrolytes, while facilitating the secretion of various factors, such as mucus, ions, and hormones [1]. However, under conditions of high-intensity exercise, particularly endurance activities, heat stress poses a significant risk to gut health by compromising this barrier. Heat exposure can disrupt epithelial integrity, increasing the likelihood of gut damage [3,4]. Upon commencing exercise, it is postulated that two molecular signalling cascades impact the GI tract: the circulatory–GI and neuroendocrine–GI pathways (Figure 1). During exercise, splanchnic blood flow is reduced as blood is redistributed peripherally to meet the metabolic demand of the working musculature through the circulatory–GI pathway [5,6]. This can alter the integrity of the junctional complexes on the intestinal barrier, causing increased permeability and allowing bacteria, pathogens, and food particles to translocate into the bloodstream, potentially triggering an inflammatory response [4,7]. Simultaneously, exercise activates the neuroendocrine–GI pathway, which stimulates the sympathetic nervous system and triggers the release of stress hormones such as cortisol and adrenaline. This hormonal response impairs gut motility and decreases nutrient absorption [5,6]. One of the primary indicators of gut damage is a measure of intestinal fatty acid-binding protein (I-FABP) levels in circulation [8]. This small (14-kD) protein, derived from enterocytes, is released quickly into circulation upon enterocyte damage [8,9]. These physiological and molecular interactions could lead to intestinal ischemia and inflammation, and have a potential secondary impact, thus impairing post-exercise muscular recovery and subsequent exercise [8,9]. These effects may present acutely or have a chronic onset, leading to longer-term health implications [9].

Endurance athletes commonly experience GI symptoms (GISs) during exercise, which tend to worsen with increasing exercise duration and exposure to challenging environmental conditions such as heat [10,11]. The incidence and severity of GISs in athletic populations varies widely, with 3–23% of athletes reporting at least one symptom during a 16 km run [10] to 96% of athletes during a 161 km ultramarathon [12]. Therefore, factors such as environmental temperature, exercise intensity and duration, training status, age, altitude, and feeding or fluid intake can significantly influence the severity and prevalence of GISs during exercise [4]. These symptoms can occur in both the upper (oesophagus and stomach) and the lower GI tract (small bowel and colon [13]), and the severity of the GI disturbance can have an adverse effect on exercise performance, potentially leading to early withdrawal from training activities or competition [13]. A significant increase in i-FABP occurred in rugby players following 60 min of vigorous endurance exercise [14] and in runners during an acute bout of high-intensity exercise [14]. Collectively, these findings demonstrate that intestinal injury can be compromised secondary to exercise onset, with more severe cases having deleterious effects on endurance performance. Nevertheless, recent evidence suggests that regular exercise and “gut training” regimes with dietary supplements may improve intestinal integrity as a function of training time, which could help mitigate the influence of gut damage during exercise [15,16,17]. Therefore, the gut damage and GISs demonstrate the capacity to adapt to a sufficient training stimulus, resulting in more favourable outcomes [5,18,19]. This adaptation process is closely linked to muscle health [20], and further understanding of how this adaptation might be facilitated and optimised among athletes is therefore required.

In addition to exercise, diet is a lifestyle factor that can influence intestinal integrity, and there is growing interest in developing effective strategies to alleviate exercise-induced GISs [4,21]. In recent years, there has been an increase in studies investigating the influence of various dietary supplements on exercise-induced gut cell damage and GISs during exercise. These include bovine colostrum [22,23], probiotics [24,25], curcumin [26], and glutamine [14]. Additionally, while some supplements, such as carbohydrates, are typically considered to worsen GISs, there is emerging evidence suggesting that certain types of carbohydrates may actually support gut function [27]. The study design of studies on dietary supplementation and exercise-induced gut damage and GISs vary, including factors such as environmental temperature (hot vs. thermoneutral [28,29], supplementation dosage [30], or supplement duration (60 min before exercise to 14 consecutive days) [22,31]). Naturally, these variations have resulted in disparities in study outcomes, necessitating a comprehensive review of the impact of dietary supplementation on exercise-induced gut cell damage and GISs. Although a recent systematic review investigated the use of dietary supplements on markers of gut damage and permeability in response to exercise [32], there were not sufficient data to meta-analyse the outcomes (search performed February 2021). Subsequent to this, more studies have been published across a wider range of dietary supplements, thereby permitting statistical evaluation. The aims of the current study were to systematically review and meta-analyse (i) the effect of exercise and dietary supplements on GISs and biomarkers of gut damage, and (ii) assess the effect of supplements targeted for GISs and gut damage on endurance exercise performance.

## 2. Materials and Methods

### 2.1. Design and Search Strategy

The search strategy was adopted based on the Preferred Reporting Items for Systematic reviews and Meta-Analyses (PRISMA) [33]. Inclusion criteria were studies in the English language; with healthy adults aged ≥18 years who had participated in an exercise trial, intervention, or habitual exercise; with a measurement of at least one gut outcome (perceptual GISs or biomarkers of gut damage); and with a randomised controlled trial conducted. Exclusion criteria were animal and in vitro studies, inability to access full-text articles, grey literature, and humans aged <18 years or those suffering from any disease. All research, regardless of the year of publication, was included, and medical subject heading (MeSH) terms were active. The review was not pre-registered. Based on the International Olympic Committee (IOC) definition, “supplements” were considered supplemental macronutrients given as sports foods or supplements alongside the standard diet. These supplements can include any additional food or food-derived substance [34]. Both acute (pre-exercise or during exercise) and chronic (multiple days) supplementation protocols were included. Supplements included ones that have been studied for their roles in enhancing gut health and muscle function, with consideration of their mechanistic effects on intestinal integrity. Endurance performance in the current study included two forms of exhaustive exercise of any modality: time to exhaustion (TTE) or time trial (TT). Any forms of exercise that were either not exhaustive or performed for <75 s were removed. Studies were included regardless of training status and participant sex. Overall effects (combinations of all modalities) and sub-groups of supplement categories were considered for the analysis.

### 2.2. Data Selection and Collection

**Figure 2:**
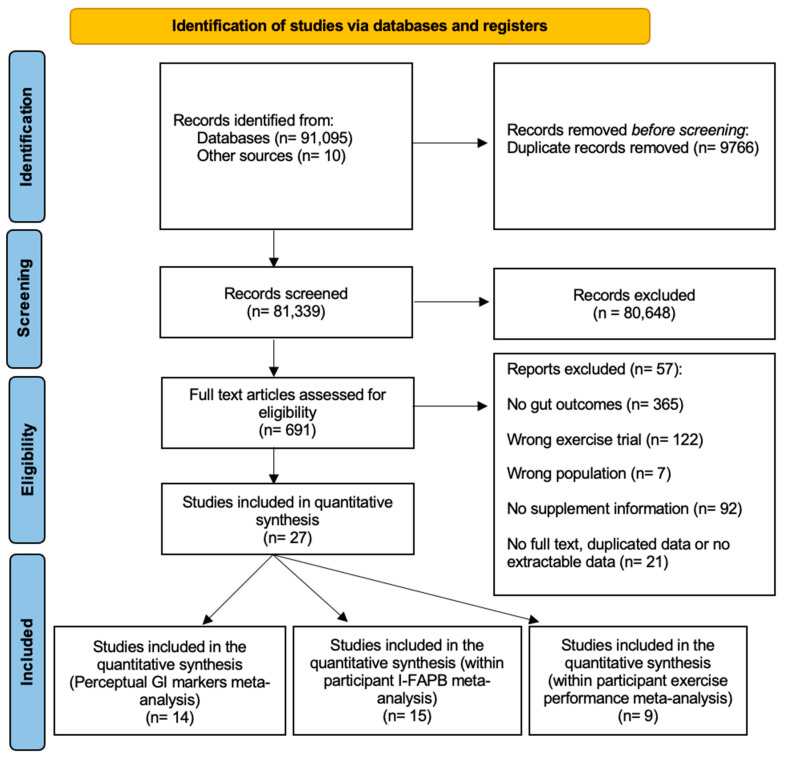
PRISMA flow diagram.

Initial screening of titles and abstracts determined suitability for inclusion. The entire article was retrieved for full-text review to determine inclusion (Figure 2). Four electronic databases were searched, including PubMed, Scopus, Science Direct, and Sports Discus. The search was carried out in December 2022, with an updated search in June 2023 and November 2024. The following search terms were intentionally broad and included “(exercise OR endurance exercise OR physical activity OR exercise training OR exercise-induced) AND (diet OR dietary patterns OR dietary factors OR dietary components OR supplement OR nutrition OR nutrients OR food OR protein OR fat OR carbohydrate OR sugar OR vegetarian OR vegan OR omnivore OR Western diet OR urban diet OR Mediterranean) OR (gastrointestinal OR gut permeability OR gut barrier OR intestin* OR gut dysfunction OR leaky gut OR gut injury OR mucosal OR splanchnic OR gut health OR GI symptoms OR GI stress, I-FAPB OR LBP OR LPS OR sCD14).” At least one of the keywords from each AND operator in the title, abstract, or keywords was required, and three authors (RA, SH, and MW) confirmed the search terms.

### 2.3. Study Selection

Retrieved articles were exported to Covidence (version 2.0, Veritas Health Innovation, Melbourne, Australia) for the screening process, where all duplicates were removed. Titles and abstracts were screened by one reviewer (RA), and the remaining articles were reviewed for full-text screening by two reviewers (RA and SH). The reasons for removal are detailed in Figure 2.

### 2.4. Risk of Bias/Data Extraction

Data were extracted to a Microsoft Excel spreadsheet. Extracted data included participant characteristics (age, sex, height, body mass, training status), study design, supplements consumed or relevant dietary data, perceptual GISs (total) or biomarkers of gut cell damage, and exercise outcomes. The risk of bias was assessed by two authors (RA and SH) following Cochrane collaboration guidelines [35].

### 2.5. Certainty of Evidence

The overall certainty of evidence across studies was evaluated following the guidelines established by the GRADE working group (Grading of Recommendations Assessment, Development, and Evaluation; [36]) as outlined on gradeworkinggroup.org.

### 2.6. Statistical Analysis

Data were extracted from the papers in the form of a mean, standard deviation (*SD*), and sample size (n) for the meta-analysis. Publicly available software (Web Plot Digitizer, V.4 6, San Francisco, CA, USA: Ankit Rohatgi, 2022) extrapolated any unreported values from figures to mean and *SD* data. Authors of the original research articles were contacted for any missing data, and if not provided, the articles were excluded from the study. If a range was reported, *SD* was calculated with the following equation [35]:

$$SD\approx \frac{max-min}{4}$$

Three meta-analyses were performed: a between-groups meta-analysis for GISs during exercise, a between-groups meta-analysis for exercise performance, and a within-groups meta-analysis for outcome scores of circulating i-FABP pre- to post exercise. Analyses were performed in RStudio and included 10, 6, and 12 comparison groups, respectively. All data were analysed with a random-effects model, with heterogeneity assessed using the I^2^ statistic. Outliers were detected using a function in RStudio, and influence on analysis was investigated. A within-groups meta-analysis was conducted for circulating i-FABP pre- to post exercise due to insufficient data between groups. Publication bias was determined using funnel plots, with Egger’s test being conducted and, subsequently, Duval and Tweedie’s trim-and-fill procedure, when indicated. Hedges’ g and 95% confidence intervals (CIs) were used to define standard mean difference (SMD) between dietary supplementation and placebo groups, and between pre- to post exercise in intervention groups. When multiple intervention groups from the same study were used in the between-study meta-analyses, effect sizes were adjusted by dividing them by the square root of the sample size in the control group [35]. A leave-one-out analysis, excluding one study at a time, was also performed to assess the role of each individual study in the pooled estimate. Pooled analysis of different supplements has previously been conducted [37,38]. Sub-analysis of the different supplements was included for both meta-analyses’. Prediction intervals were incorporated to estimate the range within which the true effect size of each supplement is expected to fall in future individual studies. Meta-regressions were also conducted to determine the effect of moderators on GISs and circulating i-FABP outcomes, as reported in each study: environmental temperature (thermoneutral or hot), duration of exercise, and mode of exercise (run, cycle and walk). Alpha (α) was set at *p* ≤ 0.05, and the thresholds for the magnitude of effects were <0.2, 0.2, 0.5, and 0.8 for trivial, small, medium, and large effects, respectively [39].

## 3. Results

### 3.1. Study Selection

The initial search retrieved 91,105 articles. Following the removal of duplicates, 81,339 remained. After title and abstract screening, removing irrelevant studies, 691 full-text studies were assessed for eligibility. Of the remaining papers, 664 were excluded based on the inclusion criteria. Searches of the selected study reference lists detected an additional 10 papers, and 27 studies were included in the meta-analysis (Figure 2).

### 3.2. Study Characteristics

The meta-analysis included 27 studies; 20 studies had a crossover design, with 7 independent group designs. Fifteen studies (18 comparison groups) assessed gut outcomes through perceived GISs using Likert scales or visual analogue scales (VAS), 15 studies (21 comparison groups) used the circulating blood marker i-FAPB to determine gut damage, and 9 studies (10 comparison groups) provided a performance measure through TTE or TT. See Table 1 for study characteristics. 

### 3.3. Participant Characteristics

A total of 495 participants were included in the meta-analysis, and the average age of was 28 ± 6 y (90% male), with no reported differences in gut responses between sex. Seventeen studies reported average height (1.75 ± 0.03 m), and 24 studies reported body mass (74.4 ± 5.1 kg), with 1 presenting BMI only [40]. All studies reported $\dot{V}$O_2peak_, and the average of all was 54.7 ± 7.2 mL·kg^−1^·min^−1^. All participants were healthy and trained, except in one study, which investigated a trained and an untrained group [5]. 

### 3.4. Environmental Characteristics

For the circulating i-FAPB meta-analysis, 50% of the studies investigated exercise in hot and/or humid environments (>23 °C [23,26,29,30,31,41,42,43,44]). The other half of the studies were conducted in a thermoneutral laboratory environment (19–23 °C [22,40,45,46,47,48,49,50,51,52]). Additionally, one study was conducted in a cold environment (−5 °C [53]). In the GIS meta-analysis, all studies, with the exception of two (35 °C, 50% relative humidity (RH) [29,54]), were conducted in a thermoneutral environment. Likewise, in the exercise performance meta-analysis, all studies were conducted in thermoneutral environments, except for one hot environment [44] and one cold environment [53].

### 3.5. Exercise Characteristics

Exercise studies used walking (n = 1), running (n = 18), cycling (n = 6), cross-country skiing (n = 1), or a combination of running and cycling (n = 1). Exercise duration ranged from 20 min to 3 h. Exercise intensity was reported as a percentage (%), $\dot{V}$O_2peak_ (n = 12, 60–90%), power (W_max_; n = 4, 55–70%), lactate threshold (n = 2, 90–100%), ventilatory threshold (n = 1, 95%), or heart rate reserve (n = 1, 50–80%). Eight studies reported a performance result, with TTE (n = 5) and distance covered (n = 3). Most studies were in a laboratory setting, with the exception of two studies that were outside in a race environment [6,7]. The exercise performance meta-analysis included four TTE and five TT outcomes across nine studies. All TTEs were performed on treadmills, except for one ski ergometer measure [6] and one outdoor track [7]. The TT involved running (n = 4 [5,8,9]) and cycling (n = 1 [10]).

**Table 1:** Summary of studies included in the meta-analysis (n = 26).

Study	Study Design	Participant Characteristics	Gut Measures		Exercise Intervention				Supplement/Diet
			i-FAPB	GISs (Mean (SD))	Mode	Duration	Intensity	Environment	
			(Mean (SD) Pre-Post)						
Rowe et al. [55]	Randomised, crossover, double-blind design	Trained males (*n* = 11) 29 ± 6 y	-	Experimental −3 (1) Control −2 (1)	Run	120 min and 5 km	$68\%\;\dot{V}$O_2peak_ SS and TT	Thermoneutral	Glucose and fructose hydrogel (180 g, 2:1 ratio)
McCubbin et al. [56]	Randomised, crossover design	Trained male endurance runners (*n* = 9) 36 ± 5 y	-	Experimental −15 (9.5) Control −18 (8.75)	Run	3 h and TTE	$60\%\;\dot{V}$O_2peak_ SS and 2 km/h increase every 3 min	Thermoneutral	Hydrogel CHO–electrolyte beverage (53 g/h maltodextrin, 37 g/h fructose)
Miall et al. [16]	Randomised, single-blinded design	Recreational runners (*n* = 18) 35 ± 8 y	-	Experimental −6.23 (0.70) Control −4.5 (0.6)	Run	120 min and 60 min	$60\%\;\dot{V}$O_2peak_ SS and TT	23 ± 1 °C, 50 ± 8%RH	CHO gel disc (30 g; 2:1 glucose–fructose)
Costa et al. [15]	Randomised, placebo, controlled design	Runners (*n* = 15 M and *n* = 10 F) 35 (32–38) y	Gel disc: 434 (158.3) –981 (362.5) Carbohydrate food: 450 (151)–1236 (618)	Experimental −50.3 (43.1) Control −43 (14)	Run	120 min and 60 min	$\mathrm{SS}\;60\%\;\dot{V}$O_2max_ and TT	Thermoneutral 50%RH	CHO (gel disc/food) (30 g; 2:1 glucose–fructose)
Oosthuyse et al. [57]	Randomised, double-blind, three-way crossover design	Trained male cyclists (*n* = 9) 38 ± 7 y	-	Experimental −2.65 (3.3) Control −0.32 (0.1)	Cycle	120 min	60%W_max_	Thermoneutral	CHO (63 g∙h^−1^ isomaltulose; 7% CHO, fructose:maltodextrin; 0.8:1 ratio, 7% CHO))
Pettersson et al. [53]	Double-blind, randomised, crossover design	Elite cross-country ski athletes (n = 6 F, 24.8 ± 5.3 y; n = 6 M, 25.6 ± 4.7)	-	Experimental −3.5 (3.3) Control −2.5 (2.7)	Cross-country skiing	120 min	$70\%\;\dot{V}$O_2peak_	−5 °C	Hydrogel CHO (132 g·h^− 1^, 1:0.8 maltodextrin:fructose)
Pugh et al. [46]	Randomised, double-blind, matched-pairs design	Runners (*n* = 20 M, 4 F)	448 (183)–1834 (1692)	-	Run	42.2 km (234 ± 38 min)	90.2 ± 9.1%LT	Thermoneutral	Probiotics (active strains *) and CHO
Pugh et al. [45]	Randomised, double-blind, placebo-controlled crossover design	Trained cyclists (*n* = 7) 23 ± 4 y	540 (144.4)–335 (189)	-	Cycle	120 min	55% W_max_	Thermoneutral	Probiotics (active strains *) and CHO
Shing et al. [54]	Randomised, double-blind, placebo-controlled crossover design	Trained runners (*n* = 10) Age 27 ± 2 y	-	Experimental −1.4 (0.2) Control −1.6 (0.3)	Run	TTE ~50 min	TTE	35 °C, 40% RH	Probiotics (active strains *)
Kekkonen et al. [50]	Randomised, double-blind design	Marathon runners (Con; n = 71, 40 (23–69) y, LGG; n = 70, 40 (22–58 y)	-	Experimental −0.4 (0.8) Control −0.6 (1.1)	Run	Habitual marathon training	-	Thermoneutral	Probiotics (LGG)
Schreiber et al. [51]	Randomised, double-blind, two-arm, placebo-controlled design	27 elite and category 1-level cyclists (28.3 ± 5.6 y)	-	Experimental −6.18 (15.37) Control −3.82 (14.02)	Cycle	TTF	85% of W_max_	Thermoneutral	Probiotics (active strains *)
Pugh et al. [30]	Randomised, double-blind, placebo-controlled crossover design	Recreationally active healthy males (*n* = 10) 24 ± 4 y	0.25 g: 314.9 (146.2)–595.7 (306.3) 0.5 g: 329.3 (187.8)–481.6 (282.8) 0.9 g: 266.6 (183.8)–488.1 (227.3)	-	Run	60 min	$70\%\;\dot{V}$O_2peak_	30 °C	Glutamine (0.25 g, 0.5 g, 0.9 g per FFM)
Osbourne et al. [29]	Randomised, double-blind, placebo-controlled crossover design	Male cyclists (*n* = 12) 32 ± 6 y	0.61 (0.15)–0.8 (0.16)	Experimental –15.2 (4.8) Control –13.2 (7.9)	Cycle	20 km TT	TT	35 °C, 50%	L-glutamine (0.9 g·kg^−1^ FFM)
Tataka et al. [48]	Randomised, double-blind, placebo-controlled crossover design	Healthy males (*n* = 16) 23 ± 3 y	1458 (1288)–1602 (1381)	-	Run	60 min	$75\%\;\dot{V}$O_2peak_	25 °C, 60%	L-cystine (0.23 g) and L-glutamine (1 g)
Ogden et al. [44]	Randomised, double-blind, placebo-controlled crossover design	Healthy males (*n* = 10) 29 ± 7 y	1.21 (0.67)–2.46 (1.17)	**-**	Run	30 min	@LT	40 °C, 40% RH	L-glutamine (0.3 g·kg^−1^ FFM)
Ogden et al. [31]	Randomised, double-blind, placebo-controlled crossover design	Healthy males (*n* = 12) 32 ± 6 y	2.16 (0.98)–4.7 (1.31)	**-**	Walk	80 min (2 × 40 min)	Fixed-intensity (6 km h^−1^ ,7% gradient)	35 °C, 30% RH	L-glutamine (0.3 g·kg^−1^)
March et al. [22]	Randomised, double-blind, placebo-controlled crossover design	Healthy males (*n* = 18) Age 26 ± 5 y	672 (394)–684 (481)	-	Run	20 min	$80\%\;\dot{V}$O_2peak_	Thermoneutral	Bovine colostrum (20 g·day^−1^)
March et al. [23]	Randomised, double-blind, placebo-controlled crossover design	Healthy males (*n* = 12) 26 ± 6 y	821.3 (147.1)–1312 (205.9)	-	Run	60 min	$70\%\;\dot{V}$O_2peak_	30.0 °C, 60% RH	Bovine colostrum (20 g·day^−1^)
McKenna et al. [43]	Randomised, placebo-controlled counterbalanced, crossover design	Healthy males (*n* = 10) 20 ± 2 y	851.35 (450.71)–1267 (521.51)	-	Run	46 min	95% VT	40 °C, 50% RH	Bovine colostrum (20 g·day^−1^)
Morrison et al. [41]	Randomised, placebo-controlled, crossover design	Trained (*n* = 7) and untrained (*n* = 7) males 22 ± 3 y	138.8 (450.7)–1267.1 (521.5)	Experimental –3.07 (4.51) Control –3.47 (3.56)	Cycle and Run	90 min total (15 min cycle, 2 × 30 min run, 15 min cycle)	Cycle – 50% HRR, 80% HRR (run 1), TT (run 2)	30 °C, 50% RH	Bovine colostrum (1.7 g·kg^−1^·day^−1^)
Szymanski et al. [26]	Randomised, double-blind, placebo-controlled crossover design	Healthy males (*n* = 6) and women (*n* = 2) 19 ± 1 y	821.3 (147.2)–1312.4 (205.9)	-	Run	60 min	$65\%\;\dot{V}$O_2peak_	37 °C, 25% RH	Meriva curcumin (500·day^−1^)
Jonvik et al. [40]	Randomised, placebo-controlled, crossover design	Well-trained males (n = 16) 28 ± 7 y	Sodium nitrate: 1037 (309)–2754.7 (11613) Sucrose: 1213 (385)–2059.4 (1096.8)	-	Cycle	60 min	70% W_max_	Thermoneutral	Sodium nitrate (800 mg), sucrose (40 g)
Kung et al. [47]	Randomised, placebo-controlled, crossover design	Cyclists (*n* = 12) 37 ± 11 y	859.4 (150.01)–1155 (213.61)	-	Cycle	45 min and TT	$70\%\;\dot{V}$O_2peak_ and TT	Thermoneutral	Dairy-based, high flavonoids (490 mg)
Taylor et al. [49]	Randomised, placebo-controlled, crossover design	Moderately trained (*n* = 16 M, 4 F 29 ± 4 y	1807 (1924)–2222.24 (3685.2)	Experimental −32.5 (38.1) Control −27.7 (33.0)	Run	70 min	$50\;\min\;@70\%\;\dot{V}$O_2 peak_ $10\;\min\;@80\%\;\dot{V}$O_2peak_ $90\;\min\;@90\%\;\dot{V}$O_2peak_	Thermoneutral	Collagen peptides (10 g·day^−1^)
Opheim et al. [28]	Randomised, placebo-controlled, crossover design	Healthy males (*n* = 19) 23 ± 3 y	-	Experimental −5.8 ± 4.5 Control −1.2 ± 1.7	Run	15 × 30 m, 35 s intervals	Self-paced (max effort)	Thermoneutral	Capsaicin (cayenne supplement)
Lassen et al. [52]	Randomised, placebo-controlled, crossover design	Healthy males (*n* = 13) and females (8) 25 ± 4 y	-	Experimental −3.5 ± 0.6 Control −3.8 ± 0.6		3.5 km TT	Self-paced (max effort)	Thermoneutral (outdoor)	Sodium bicarbonate (0.3 g/kg BM)

Gastrointestinal symptoms, GI; ventilatory threshold, VT; lactate threshold, LT; heart rate reserve, HRR; relative humidity, RH; peak oxygen uptake, $\dot{V}$O_2peak_; time to exhaustion, TTE; steady state, SS; meters, m; minute, min; seconds, s; fat-free mass, FFM; time to fatigue, TTF; maximum wattage, W_max_; carbohydrate, CHO; males, M; females, F; thermoneutral, 19–23 °C; * active probiotic strains: Lactobacillus acidophilus L60, L. acidophilus CUL21, Bifidobacterium bifidum CUL20, Bifidobacterium animalis subsp. Lactis CUL34, Lactobacillus rhamnosus GG, LGG.

### 3.6. Risk of Bias

**Figure 3:**
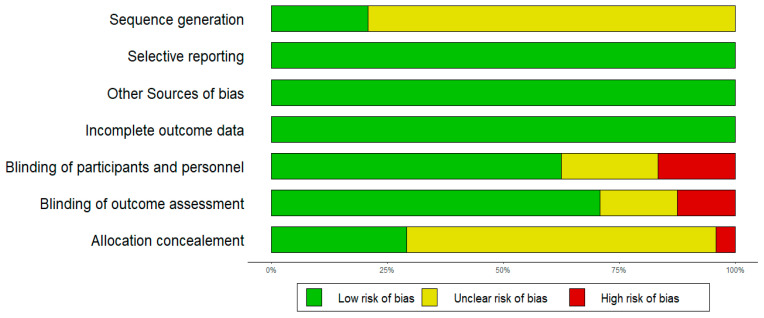
Risk of bias.

The studies generally had an “unclear” or “low” risk of bias (Figure 3), with 20 of the studies not stating a randomisation process and two studies not implementing a blinded study design. Sequence generation was “unclear” for 21 studies.

### 3.7. Meta-Analysis

**Figure 4:**
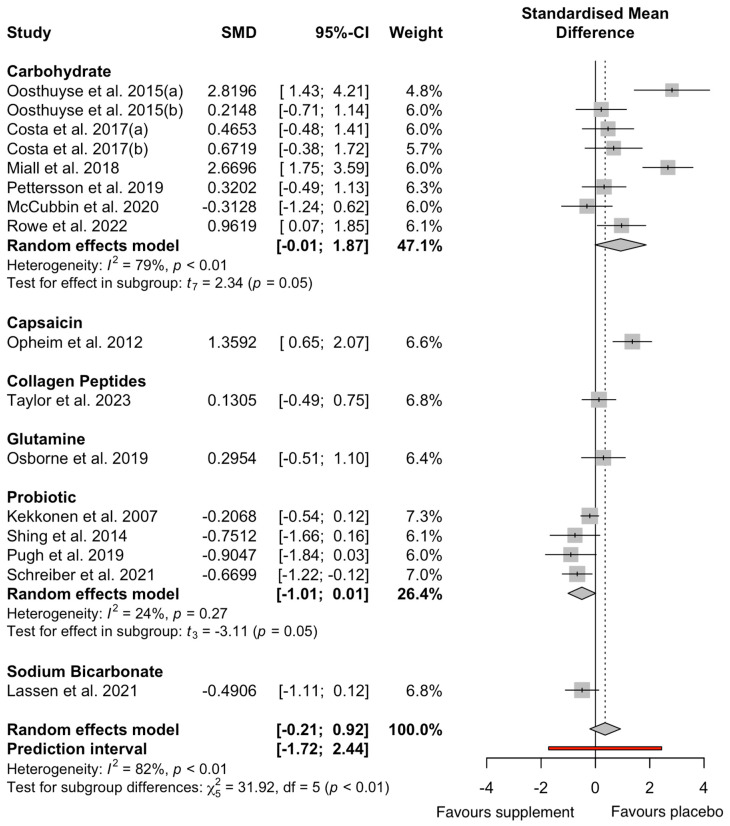
Effect of dietary supplement on GISs during exercise. Data from references [15,16,28,29,46,49,50,51,52,53,54,55,56,57].

**Figure 5:**
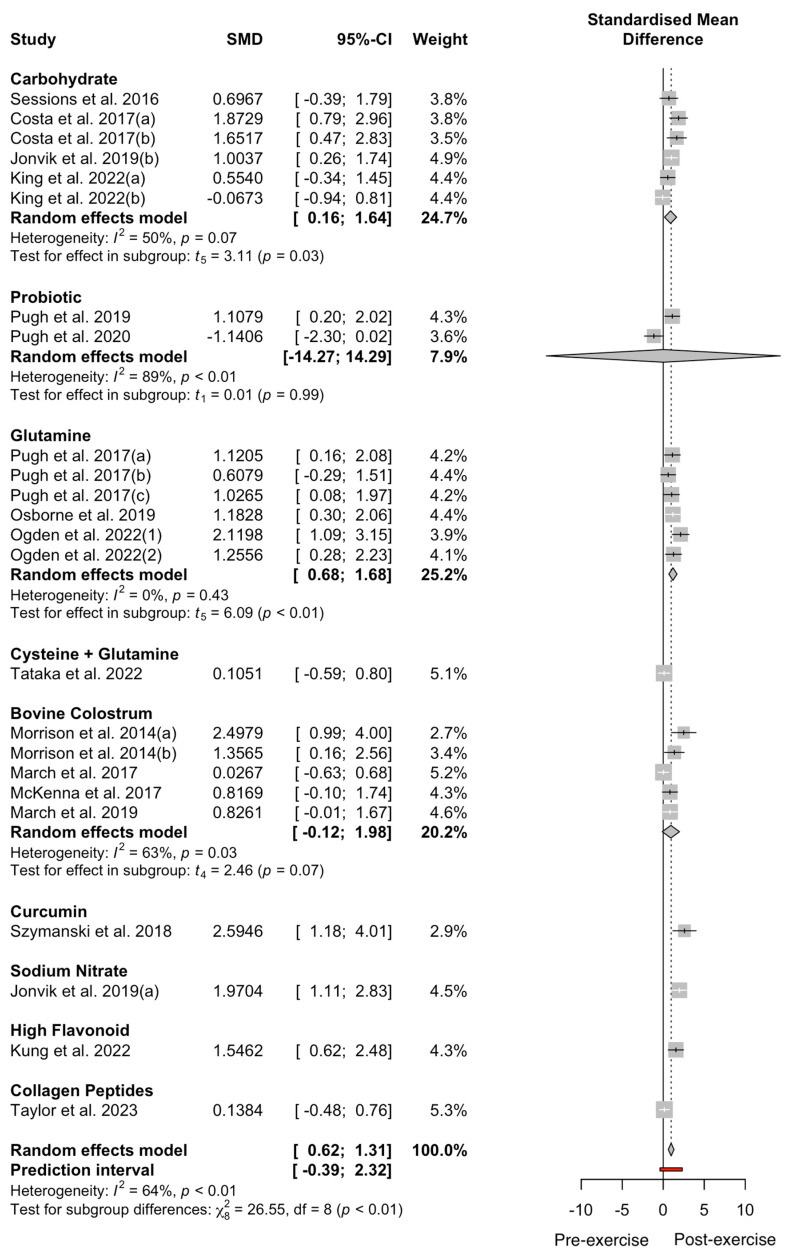
Effect of dietary supplement on circulating i-FAPB pre- to post exercise. Data from references [15,22,23,26,29,31,40,40,41,42,43,44,45,46,47,48,49,58].

**Figure 6:**
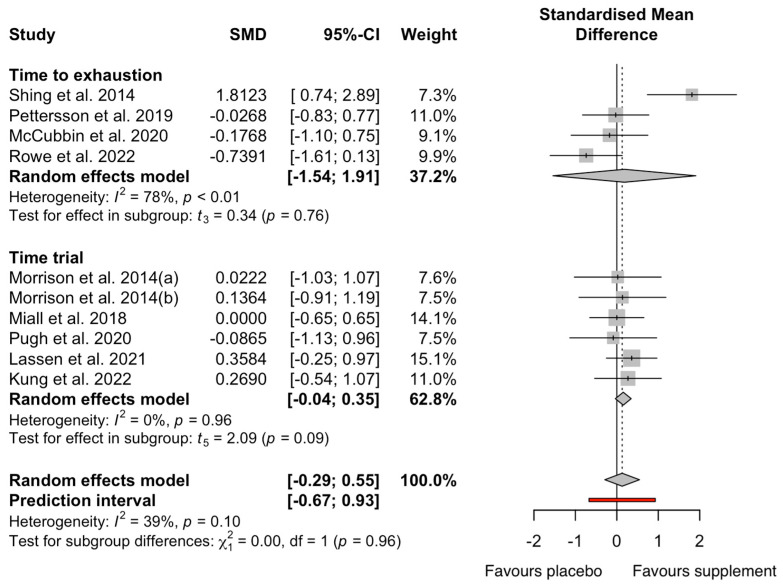
Effect of dietary supplement on exercise performance by exercise modality. Data from references [16,41,45,47,52,53,54,55,56].

The results of the GIS meta-analysis (n = 14) are presented in Figure 4. Overall, there was no effect of the pooled supplement interventions on self-reported GISs during exercise compared to the placebo (Hedges’ g = 0.42, 95% CI −0.21: 0.92, *p* = 0.14). The analysis revealed high heterogeneity, with an I2 statistic of 82%. The pooled data showed a significant increase in i-FABP concentrations pre- to post exercise, irrespective of supplements (∆106%; Hedges’ g = 1.01, 95% CI 0.63; 1.38, *p* = 0.01), with the I^2^ statistic demonstrating moderate heterogeneity (I^2^ = 62%; Figure 5). There was no effect of the pooled supplement interventions on exercise performance compared to the placebo (Hedges’ g = 0.09, 95% CI −0.41; 0.46, *p* = 0.67). The analysis revealed moderate heterogeneity (I^2^ = 43%; Figure 6).

### 3.8. Subgroup Analysis

Among the different subgroups, supplements with a positive skew presented with more GISs during exercise and, thus, did not demonstrate a favourable effect. Probiotics presented a moderate significant effect (Hedges’ g = −0.62, 95% CI −1.01 to 1.01, *p* = 0.05), favouring the supplement. Sodium bicarbonate presented a non-significant moderate effect (Hedges’ g = −0.50, 95% CI −1.11 to 1.12, *p* = 0.05), also favouring the supplement. However, capsaicin (Hedges’ g = 1.36, 95% CI 0.65 to 2.07, *p* = 0.31) and carbohydrates (Hedges’ g = 0.93, 95% CI −0.06 to 1.48, *p* = 0.07) had large non-significant effects, favouring the placebo. Glutamine (Hedges’ g = 0.30, 95% CI −0.509 to 1.100, *p* = 0.27) and collagen peptides (Hedges’ g = 0.13, 95% CI −0.49 to 0.75, *p* = 0.24) exhibited a small non-significant effect without a directional change.

Among the different subgroups, supplements with a positive skew presented with more i-FABP pre- to post exercise and thus did not demonstrate a favourable effect. Glutamine (Hedges’ g = 1.18, 95% CI 0.68; 1.68, *p* = 0.001) and carbohydrates (Hedges’ g = 1.38, 95% CI 0.19; 2.58, *p* = 0.04) had a large significant effect. Bovine colostrum (Hedges’ g = 0.93, 95% CI −0.12; 1.98, *p* = 0.07), sodium nitrate (Hedges’ g = 1.97, 95% CI 1.10; 2.83, *p* = 0.19), high flavonoids (Hedges’ g = 1.55, 95% CI 0.61; 2.47, *p* = 0.99), collagen peptides (Hedges’ g = 0.14, 95% CI −0.48; 0.76, *p* = 0.26), combined cysteine and glutamine (Hedges’ g = 0.11, 95% CI −0.58; 0.79, *p* = 0.18), and curcumin (Hedges’ g = 2.59, 95% CI 1.18; 4.01, *p* = 0.18) presented large non-significant effects, not favouring the supplement. However, probiotics (Hedges’ g = 0.01, 95% CI −14.27; 14.29, *p* = 0.99) presented a large CI and overall, a trivial non-significant effect was observed.

Time to exhaustion (Hedges’ g = 0.18, 95% CI −1.54; 1.91, *p* = 0.77) and TT groups (Hedges’ g = 0.15, 95% CI −0.04; 0.35, *p* = 0.09) had small non-significant effects with wide CIs. Sub-group analysis for supplement categories were not significant for performance (*p* = 0.96). Carbohydrates (Hedges’ g = −0.06, 95% CI −0.78; 0.77, *p* = 0.72), flavonoids (Hedges’ g = 0.36, 95% CI −0.25; 0.97, *p* = 0.33), and sodium bicarbonate (Hedges’ g = 0.27, 95% CI −0.54; 1.07, *p* = 0.55) had small non-significant effects, and probiotics (Hedges’ g = 0.82, 95% CI −12.20; 13.91, *p* = 0.61) showed a large non-significant effect.

### 3.9. Meta-Regression

For both GISs and circulating i-FABP meta-analysis, there were no moderating effects of any extracted variables and their interactions on GISs during exercise and circulating i-FABP pre- to post exercise (Table 2). The analysis showed residual heterogeneity (τ^2^ = 0.384, *p* = 0.001), indicating that there was still unexplained variability in the effect sizes among the studies. The overall model accounted for 63% of the heterogeneity in the effect sizes. No meta-regression was required for the exercise performance meta-analysis.

### 3.10. Publication Bias

**Figure 7:**
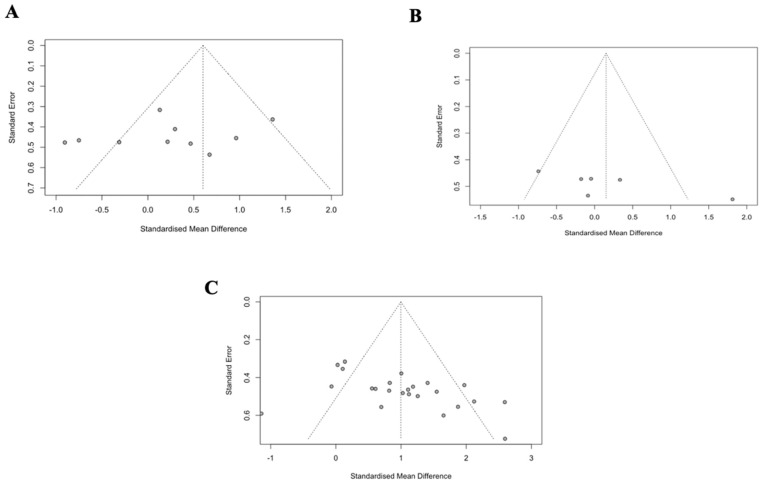
Publication bias for (**A**) exercise-induced GISs, (**B**) circulating i-FABP, and (**C**) exercise performance.

One outlier was detected from the GIS meta-analysis; however, the Egger’s test revealed no evidence of publication bias (*p* = 0.502, Figure 7A). Several outliers were observed in the circulating i-FABP meta-analysis, owing to the large effects certain supplements seem to have on post-exercise circulating i-FABP. The Egger’s test indicated publication bias (*p* = 0.012; Figure 7B), and the Duval and Tweedie’s trim-and-fill procedure was conducted, but no meaningful changes in the data were observed. The exercise performance meta-analysis showed no publication bias despite one outlier (*p* = 0.075; Figure 7C); however, it is recognised that this meta-analysis only included a small number of comparisons (n = 7). In addition, in the leave-one-out analysis, none of the studies impacted the results.

### 3.11. Grading of Evidence

GRADE assessment was conducted for each primary outcome—i-FABP, GISs, and exercise performance—and additionally by supplement subgroup (Table 3). Overall, the quality of the evidence was low or very low.

## 4. Discussion

The main findings of the current meta-analyses were that circulating i-FABP significantly increased pre- to post exercise (Δ106%; Hedges’ g = 1.01, *p* = 0.01). There were no significant reductions in i-FABP pre- to post exercise following any supplement categories. Notably, there was a wide confidence interval for probiotics, with a trivial non-significant effect (Hedges’ g = 0.01, *p* = 0.99), contributing to the lower i-FABP concentrations. While glutamine and carbohydrates had large significant effects for circulating i-FABP pre- to post exercise, no studies observed a clinically meaningful threshold of Δ ≥ 1000 pg·mL^−1^, suggested to indicate practical relevance [11,12]. Among all the supplement categories, only probiotics significantly lowered GISs (Hedges’ g = −0.83, *p* = 0.05), while the overall supplement categories had no significant effect on GISs during exercise (*p* = 0.15; Figure 4). The 95% CI for the overall effect of supplements on GISs ranged from −0.17 to 1.02, suggesting an equal likelihood of no effect or a small to moderate effect. This imprecision highlights the uncertainty and underscores the need for further research with more precise estimates. Finally, supplement categories did not significantly affect TTE (*p* = 0.77; Figure 6) or TT (*p* = 0.62) performance. While some trends were observed, the wide CIs and lack of statistically significant findings suggest that the results may not have a substantial clinical or practical relevance.

### 4.1. Probiotics

The anti-inflammatory role of probiotics is the mechanism considered to reduce exercise-induced intestinal integrity and help to promote short-chain fatty acid production, thereby enhancing gut barrier function [59,60]. Two studies [46,54] investigated the effects of probiotics on GISs (Hedges’ g = −0.83; *p* = 0.05); there was a consistent reduction in GISs across studies, and a moderately significant effect was observed at the sub-group level. Two studies [9,14] reported on circulating i-FABP (Hedges’ g = 0.01, *p* = 0.99) and demonstrated opposing effects, presenting a trivial non-significant effect on circulating i-FABP. Time to exhaustion performance was also affected inconsistently, with both positive and negative outcomes following probiotic supplementation [45,54], resulting in a large non-significant effect on performance (Hedges g’= 0.82, *p* = 0.61).

The observed discrepancy in circulating i-FABP between studies may be attributed to the lower-intensity exercise (50% W_max_) or the exercise mode (i.e., cycling [45]). Exercise such as cycling and swimming may not consistently induce a rise in circulating i-FABP compared to running, which can cause increased mechanical strain and intestinal ischemia [61,62,63,64]. Additionally, the use of intra-workout fuelling, such as carbohydrate gels for runners (66 g·h^−1^ [46]) and maltodextrin drinks for cyclists (88.2 g·h^−1^; [45]), may have influenced post-exercise circulating i-FABP levels. As previously demonstrated [27], carbohydrate ingestion blunted the i-FABP response during exercise. Immediately post exercise, no significant differences (*p* > 0.05) in total reported GISs were observed between the probiotic and placebo groups, reporting median scores of 13 and 15, respectively [46]. Notably, during the final third of the race, the mean GIS score was significantly (*p* = 0.01) lower in the probiotic group than in the placebo group (3.5 and 6.1, respectively). Exercise performance can be directly influenced by GISs. For example, an athlete may feel the need to reduce exercise intensity or cease exercising fully owing to increased GISs [11,13]; however, findings in the meta-analysis are contrasting. Pugh and authors [45] observed a lower cycling TT performance (*p* = 0.71) for the probiotics group (301 ± 74 s) compared to the placebo (308 ± 69 s), while during a running TTE, a significant increase in performance (*p* = 0.03) for the probiotics group (37:44 ± 2:42 min) compared to the placebo (33:00 ± 2:27 min) was observed [54]. While probiotics may have had a beneficial effect, their overall impact on GISs might have been limited due to the already low baseline levels of GISs observed across studies [50,54]. Furthermore, GISs were recorded using various scales, such as a 0–100% visual analogue scale [51], a 0–10 Likert scale [46], a 1–4 scale [54], and counting the incidence of GISs [51]. Thus, the inconsistency in measurement methods could have contributed to the mixed results, making it challenging to draw definitive conclusions about the efficacy of probiotics in reducing GISs. Standardised assessment tools are necessary for more reliable and comparable results in future research, including a validated scale and uniform symptom-reporting protocols. 

### 4.2. Glutamine and Cysteine

Glutamine is a non-essential amino acid that aids the proliferation and differentiation of intestinal epithelial cells, which are responsible for the absorption of nutrients and protection against harmful substances [65]. Four studies [29,30,31,44] observed a rise in i-FABP pre- to post exercise, with a large significant effect (Hedges’ g = 1.18, *p* = 0.001; Figure 5), indicating minimal effect for reducing post-exercise circulating i-FABP. One study measured GISs (Hedges’ g = 0.30, *p* = 0.27 [29]), showing no effect. Glutamine supplementation (0.5 g·kg^−1^ and 0.9 g·kg^−1^) reduced circulating i-FABP during a 60 min run in a hot environment (30 °C) but with no significant effect [30]. However, 0.9 g·kg^−1^ of glutamine did not change circulating i-FABP or exercise performance [29]. Similarly, ingestion of 0.3 g·kg^−1^ of glutamine before an incline walk (6 km·h^−1^, 7% incline) and a run in a hot environment (35 °C and 40 °C) did not affect circulating i-FABP (*p* > 0.05 [31,44]). Glutamine ingestion ranged from 60 min [29,31,44] to 120 min [30] before exercise. Therefore, differences in dosing and the time required for glutamine to optimally affect cell proliferation, exercise mode or duration, environmental temperature, and the likelihood of running for more than 1 h, especially at higher intensities (>70% $\dot{V}$O_2peak_) and in hot environments, may cause a greater rise in i-FABP and GISs compared to cycling, walking, or swimming [4].

Cysteine, another non-essential amino acid, serves as the primary precursor for glutathione, a powerful antioxidant that defends cells against oxidative damage, which induces a rise in circulating i-FABP [66]. One study [48] measured the effects of a combined supplement of glutamine and cysteine on i-FABP but reported no effects (Hedges’ g = 0.11, *p* = 0.18). However, it is important to emphasise that glutamine- or cysteine-only groups were not investigated. Thus, further research is warranted to understand the potential benefits of co-ingestion and the specific contributions of glutamine and cysteine supplementation on attenuating intestinal damage during exercise.

### 4.3. Bovine Colostrum

It is hypothesised that bovine colostrum can improve the integrity of the gut wall by increasing the production of tight junction proteins, which aid in preventing the translocation of bacterial toxins into the bloodstream [67]. Within the meta-analysis, four studies [22,23,41,43] examined the effects of bovine colostrum on circulating i-FABP, and a large non-significant effect was observed (Hedges’ g = 1.10, *p* = 0.29), indicating an overall non-favourable increase in i-FABP. However, only one study [40] was included in the exercise performance meta-analysis, and no significant differences in 1 h cycling TT was observed following 14 days of bovine colostrum supplementation (20 g·day^−1^) compared to the placebo. When examining individual studies [22,23], a blunting effect of 20 g·day^−1^ on i-FABP during a 20 min run at 22 °C and a 60 min run at 30 °C compared to the placebo was reported, respectively. Following a run (46 ± 8 min) at 40 °C, this effect appeared to be absent [43], possibly due to the exacerbation of splanchnic hypoxia and increased epithelial cell damage in a hot environment [22,23,41,43]. It is worth highlighting that i-FABP levels tend to be higher in hot environments compared to thermoneutral conditions [23,68], suggesting that the reported benefits of bovine colostrum may not extend to greater environmental temperatures, where heightened thermal gain exacerbates heat strain [43]. Additionally, only one study [22] accounted for the individual losses in plasma volume during hot exercise by statistically adjusting the i-FABP concentrations, which is recommended for all plasma biomarkers in such circumstances [69]. The absence of such corrections could introduce uncertainty and bias, altering the narrative surrounding i-FABP responses to thermal stress, thereby impacting the overall interpretation of the benefits of bovine colostrum supplementation [70].

Circulating i-FABP levels were significantly lower in untrained ($\dot{V}$O_2peak_ < 50 mL·kg^−1^·min^−1^, <3 days/week) than in trained participants ($\dot{V}$O_2peak_ ≥ 60 mL·kg^−1^·min^−1^, ≥6 days/week) (*p* < 0.01 [41]). However, bovine colostrum supplementation had no effect (1.7 g·kg^−1^ day^−1^) for either group. Therefore, bovine colostrum of 20 g·day^−1^ for 14 days may have blunting effects on circulating i-FABP during exercise in a thermoneutral environment. However, its effectiveness may vary with exercise intensity, duration, heat exposure, and training status, which are important considerations for future studies.

### 4.4. Carbohydrates

Studies supplementing with carbohydrates demonstrated a large non-significant increase in circulating i-FABP from pre- to post exercise (Hedges’ g = 0.93, *p* = 0.07) and a large yet more variable (and thus non-significant) increase in GISs compared to placebo groups (Hedges’ g = 0.89, *p* = 0.06). For exercise performance, four studies supplementing with carbohydrates were included and no significant changes in TTE or TT performance were observed (*p* = 0.64). The wide CI suggests substantial variability in responses and indicates imprecision in the estimate of the true effect size. Despite this, carbohydrate supplementation is a well-known ergogenic aid for endurance performance [71], yet the suggested maximal rates (90 g·h^−1^) of multi-transportable exogenous carbohydrate ingestion during exercise are not always well tolerated [15,16], which has led to several investigations regarding the optimal dose and form of carbohydrates ingested during exercise. Diverse saccharides, including glucose, fructose, and sucrose, can modulate intestinal permeability and damage by interacting with epithelial cells, altering tight junction integrity, and influencing paracellular transport pathways within the intestinal lining [32,72]. Whilst these mechanisms might explain the higher i-FBAP concentrations reported, it appears that the form of supplementation might influence the effect upon i-FABP concentration. The absence of a breakdown of carbohydrate forms (Figure 4 and Figure 5) was due to a lack of available data, highlighting the need for future studies to specifically investigate the distinct impact of individual carbohydrate supplements for intestinal injury. Post-exercise i-FABP was significantly lower when ingesting a drink of solely sucrose (20 g) compared to the placebo (*p* < 0.05 [40], while [15] showed that consuming carbohydrates in whole-form food elicited a numerically higher circulating i-FABP (1236 pg·mL^−1^) post exercise compared to the gel disc group (981 pg·mL^−1^). The ability to digest whole-form food during exercise will decrease, owing to the body’s redirection of blood flow to the working muscles and a reduced capacity for efficient digestion, which could lead to increased GI distress. As a result, the delivery mode and specific form of the supplement become crucial factors influencing GI characteristics, substrate delivery, absorption rate, and performance effects [73].

Carbohydrate ingestion revealed opposing responders for the prevalence of GISs during exercise, and the carbohydrate form could play a pivotal role in driving such responders. The findings by [57] revealed one of the largest GIS responders in the sub-group analysis, where GISs were greater during exercise when ingesting a 7% carbohydrate solution of slowly absorbed isomaltose compared to a quickly absorbed 0.8:1 fructose:maltodextrin. The authors hypothesised that this directly affected TT performance, which was lower following the isomaltose ingestion [57]. Similarly, the findings of [16] showed higher responders with ingestion of a 2:1 glucose–fructose gel disc (90 g·h^−1^) during a 2 h run (60% $\dot{V}$O_2max_). The original research observed that GISs were induced within 30 min and steadily increased, although not significantly (*p* > 0.05), while the gel disc group had more symptoms compared to the placebo, but GISs did not influence performance, as both groups covered the same distance (11.7 km [16]). Notably, while not fully elucidated, habitual diets may have an influence on this responder paradigm [58].

Hydrogel-based sports foods have been shown to improve gastric emptying, enhance nutrient delivery, and reduce GISs during exercise [72]. A carbohydrate–electrolyte hydrogel solution induced the fewest GISs compared to a nutrient-matched placebo, with no significant differences (*p* > 0.05) observed for exercise performance [56], and an 18% carbohydrate–hydrogel drink did not significantly alter GISs compared to a placebo [53]. Additionally, hydrogel ingestion of a glucose:fructose mixture presented significantly fewer exercise-induced GISs and improved 5 km TT performance compared to a non-hydrogel solution (*p* < 0.05 [55]). Notably, participants fasted before the trial, which may have influenced the outcomes. It is postulated that consuming a meal before exercise may mitigate splanchnic hypoperfusion during the initial stages of exercise, meeting the body’s digestive demands and improving carbohydrate absorption, thus reducing GISs [27,32]. The addition of a hydrogel carbohydrate–electrolyte solution consumed during endurance running presented mixed results experimentally for GISs and exercise performance. Further research is warranted to explore the specific contexts and mechanisms underlying the impact of hydrogel ingestion on GISs, biomarkers of gut damage, and performance.

These results indicate that carbohydrate ingestion during exercise can induce varying degrees of GISs and circulating i-FABP levels. While the effects of carbohydrate supplementation on GISs and i-FABP levels vary across studies, it is important to acknowledge the potential impact of these symptoms for athletes, as carbohydrates are a necessary fuel source. Some findings suggest that hydrogel ingestion composed of glucose:fructose (2:1 ratio) may lead to lower exercise-induced GISs compared to the non-hydrogel solution [55]. Additionally, consuming a meal before exercise may improve saccharide absorption into the gut and lessen GISs. The choice of delivery mode, such as the form and composition of the supplement or food, can affect how efficiently nutrients are absorbed in the gut and may contribute to GI distress during exercise [73,74], and consuming whole food sources may increase i-FABP more than other forms (gels or solutions) due to the speed of digestion and absorption. Slowly absorbed carbohydrates, such as isomaltose, lead to greater GISs than quickly absorbed carbohydrates, such as fructose:maltodextrin. Furthermore, incorporating gut training [75] into the multi-pronged strategy to increase carbohydrate availability may alleviate gut damage and enhance the tolerability of increased carbohydrate intake during prolonged high-intensity exercise for athletes. It is crucial not to overlook the potential of mitigating symptoms associated with carbohydrate ingestion, as it is an essential nutritional strategy for athletes.

### 4.5. Other Dietary Supplements

Curcumin, a natural compound found in turmeric, presented the largest effect on pre-to-post-exercise changes in i-FABP of any of the supplements [26], although this was not significant (Hedges’ g = 2.59, *p* = 0.18). Notably, the rise in i-FABP levels could be attributed to the exertional heat stress posed during the 60 min run under hot conditions (37 °C), resulting in a marked elevation in response. However, the original research did report a significant reduction in circulating i-FABP compared to the placebo [26]. Previous reports have indicated that curcumin has been shown to enhance intestinal barrier function by downregulating LPS-stimulated IL-1β production in intestinal epithelial cells, leading to improved maintenance of the tight junctions [76]. The effect of curcumin appears to be specific to enterocyte cells, suggesting curcumin can reduce inflammation despite low bioavailability in the epithelium [76,77]. However, only one study was included within the meta-analysis, and more research is required under different exercise demands to confirm curcumin’s potential to reduce circulating markers of gut damage such as i-FABP.

Flavonoids, a prominent subclass of polyphenols, are abundant in fruits and vegetables [78]. A dairy-based high-flavonoid drink was postulated to improve gut damage during exercise through anti-inflammatory and antioxidative actions [47]. Flavonoids presented a large, non-significant effect on circulating i-FABP pre- to post exercise changes (Hedges’ g = 1.55, *p* = 0.99), and no differences in performance (*p* > 0.05; Figure 6). The conducted trial encompassed a 45 min submaximal cycling trial (70% $\dot{V}$O_2max_), succeeded by a 15 min maximal effort in a controlled laboratory environment (23 °C) with no significant differences in cycling distance (low flavonoids: 7.3 ± 0.3 km, high flavonoids: 7.4 ± 0.3 km, *p* = 0.546). There were no differences in i-FABP between the high- and low-flavonoid groups, indicating no benefit on gut damage during exercise. The authors did not indicate whether habitual diets during the supplementation period were recorded, which represents a potential limitation that could introduce variability in dietary intake and potentially influence outcomes. The substantial increase in i-FABP represents an intriguing response within a laboratory-based cycling trial, particularly when juxtaposed against trials featuring more rigorous demands.

Nitric oxide is vital as a vasodilator, controlling microvascular permeability [40]. However, due to its short-lived nature, direct application to the gut is challenging. One study [40] was included in the meta-analysis for sodium nitrate (Hedges’ g = 1.97, *p* = 0.193), presenting a large, non-significant effect on circulating i-FABP pre- to post exercise. This was the first study to investigate whether the role of sodium nitrate, a precursor for nitric oxide synthesis, may enhance splanchnic blood flow to enhance GI function during exercise in humans. However, following acute ingestion of sodium nitrate (800 mg) 150 min before a 60 min cycle (70% W_max_), no significant difference in circulating i-FABP was reported (*p* > 0.05). Although it has been postulated that an increase in nitric oxide availability may increase splanchnic blood flow and reduce GI discomfort during exercise [40], more research is required to understand the effects of nitrates on gut damage and GISs.

One study was also included for collagen peptides, presenting a small, non-significant effect on GISs post exercise (Hedges’ g = 0.13, *p* = 0.24) [49] and circulating i-FABP pre- to post exercise (Hedges’ g = 0.14, *p* = 0.26). Both in vitro studies and studies on mice have shown promising results when using collagen peptides to prevent intestinal barrier dysfunction by maintaining the expression of tight junction proteins and reducing inflammation [79,80]. Following one week of supplementation of collagen peptides (10.·g·day^−1^), circulating i-FABP and GISs post exercise were not attenuated [49]. The authors excluded participants with prior GI complaints, potentially limiting the study’s ability to detect more pronounced supplement effects. As this was the first study, further research is required to better understand the role of collagen peptides on gut damage during exercise, including investigations into the optimal timing of ingestion and dosage.

Capsaicin is an active component in hot peppers and has shown potential effects on muscle contraction, pain perception, and inflammatory response in vitro and in animal models [81,82]. However, capsaicin ingestion on exercise performance remains unclear [28]. Capsaicin (Hedges’ g = 1.36, *p* = 0.06) exhibited the largest effect size in the GIS meta-analysis, indicating a higher prevalence of symptoms. This outcome is indicative of a negative impact on performance and suggests the potential for exercise cessation due to heightened discomfort. Ingestion of capsaicin (3 g·day^−1^ cayenne for 7 days) before a repeated sprint trial led to moderate and severe GISs such as nausea, cramping, and burning bowel movements, with several participants withdrawing from the study [28]. Capsaicin activates the TRPV1 receptor in the GI tract, leading to increased sensitivity of pain fibres and gastric acid production, which can cause discomfort during exercise. These effects are exacerbated by reduced blood flow to the GI tract during exercise [83]. Capsaicin did not improve exercise performance and induced negative GISs; therefore, supplementation with capsaicin is not recommended during exercise [28].

Sodium bicarbonate (NaHCO₃) is a buffering agent used to counteract exercise-induced acidosis by increasing blood bicarbonate levels and extracellular buffering capacity, thereby delaying fatigue during high-intensity exercise [84]. In the meta-analysis, sodium bicarbonate in one study [52] presented a non-significant moderate effect size (Hedges’ g = −0.50, 95% CI −1.11 to 1.12, *p* = 0.05), favouring supplementation for GISs. Ref. [52] investigated sodium bicarbonate supplementation in elite runners, demonstrating a performance improved by 6 s; however, no significant differences in GISs were reported following a 3.5 km TT run. The relatively short distance may not have elicited conditions severe enough to amplify GISs. Sodium bicarbonate’s adverse effects on GISs are proposed to arise from its osmotic and alkalising properties, which can disrupt GI function, particularly under reduced blood flow during exercise [85]. While sodium bicarbonate has potential performance benefits, its supplementation may require careful timing and individualised protocols to mitigate GI distress and optimise outcomes.

## 5. Conclusions

Increased GISs during exercise may negatively impact athletic performance, potentially leading to early withdrawal from training activities or competition [13]. Increased circulating i-FABP post exercise indicates intestinal epithelial cell damage and, while not a direct cause, is associated with an increased risk of inflammation, endotoxemia, and potential impairments in recovery and nutrient absorption [4,86]. Furthermore, sarcopenia may influence these outcomes by exacerbating gut health issues related to muscle loss during intense physical activity, identified through ultrasound [87]. However, further research is required to fully elucidate the relationship between i-FABP levels and specific clinical outcomes. Fifty percent of the trials in this meta-analysis were conducted in hot conditions, which can exacerbate heat-related stress on the gut. However, considering the prevalence of exercise sessions lasting more than 1 h among athletes, the majority of exercise protocols retrieved were relatively short, with only 11 trials (46%) exceeding 1 h and 4 (17%) exceeding 2 h, thus limiting the current understanding of certain supplements on gut damage during prolonged exercised. Further, the quality of the evidence was considered low or very low, and notable limitations were recognised across the included studies, including a lack of control for plasma volume, the use of varied tools to measure GISs that were not validated or reliable, and inconsistencies in carbohydrate feeding protocols. These problems contribute to variability in the results and may affect the overall interpretation and comparability of the findings. Future research should address these methodological considerations.

The influence of various dietary supplements on exercise-induced changes in GISs and circulating i-FABP levels is complex and multifaceted. Probiotics, known for their potential to enhance gut health and immune function, presented conflicting findings, possibly due to errors in experimental design, such as carbohydrate feeding, lack of plasma volume corrections, and lack of clinically relevant thresholds when assessing differences [19]. Glutamine and co-ingestion of glutamine and cysteine displayed a small, non-significant effect on i-FABP levels, with conflicting findings possibly due to dosing, exercise mode, and training status, requiring further investigation. Bovine colostrum’s impact on i-FABP was inconsistent, with a large non-significant effect, possibly influenced by exercise intensity, duration, and thermal stress. Carbohydrate supplementation, a well-known ergogenic aid, induced a significant increase in i-FABP, reflecting the intricacies of carbohydrates’ interaction with gut cell damage. Notably, ingestion of hydrogel composed of glucose:fructose (2:1 ratio), consuming a pre-exercise meal, and incorporating gut training regimes were identified as strategies to mitigate GISs. The included dietary supplements had minimal clinical relevance for reducing exercise-induced gut damage, as indicated by i-FABP levels not reaching the clinically meaningful threshold of Δ ≥ 1000 pg/mL [61,62]. Probiotics and certain carbohydrate formulations that reduce GISs may enhance performance by minimising gut discomfort, while supplements like capsaicin, which increase GISs, can negatively affect performance. Thus, considering GISs is crucial when selecting supplements for athletes. However, the magnitude of GIS responses was generally not clinically significant enough to impact exercise workload or require exercise cessation, except in cases like capsaicin, where increased GISs did have a notable effect on performance. Nevertheless, these findings highlight the importance of optimising personalised carbohydrate strategies, selecting appropriate supplements, and exploring dietary patterns to manage and prevent exercise-induced GISs and biomarkers of gut cell damage (e.g., i-FABP) while enhancing athletic performance. Athletes should optimise exercise nutrition through personalised strategies, carefully selected supplements, gut training, and individualised approaches to minimise GISs and support performance. Further research in this area holds promise for developing effective strategies and supplements to support GI health during exercise, but much more research is needed, as well as on alternatives to those cited here.

## Figures and Tables

**Table 2:** Meta-regression outcomes.

Moderator	Pre-Post Circulating i-FAPB Response	GIS Response
Duration of exercise	β = 0.022, *p* = 0.448 (*n* = 21)	β = −0.02, *p* = 0.582 (*n* = 13)
Environmental temperature	β = −0.447, *p* = 0.755 (*n* = 21)	β = 1.977, *p* = 0.154 (*n* = 13)
Exercise mode	β = 0.144, *p* = 0.889 (*n* = 21)	β = 1.041, *p* = 0.390 (*n* = 13)
Exercise mode × Environmental temperature	β = 0.164, *p* = 0.819 (*n* = 21)	β = 1.366, *p* = 0.901 (*n* = 13)
Exercise duration × Environmental temperature	β = −0.009, *p* = 0.564 (*n* = 21)	β = 0.011, *p* = 0.751 (*n* = 13)

**Table 3:** GRADE assessment of evidence quality for supplements and outcomes.

Outcomes	No. of Studies	Risk of Bias	Inconsistency	Indirectness	Imprecision	Publication Bias	Quality of Evidence
**Gastrointestinal Symptom Outcomes**							
Carbohydrates	7	Not serious ^a^	Very serious ^b^	Not serious ^d^	Very serious ^e^	Serious ^g^	⊕OOO Very low
Capsaicin	1	Not serious ^a^	Not serious ^c^	Not serious ^c^	Very serious ^e^	Not assessed ^f^	⊕⊕OO Low
Probiotics	2	Not serious ^a^	Not serious ^d^	Not serious ^d^	Very serious ^e^	Very serious ^h^	⊕⊕OO Low
Collagen peptides	1	Not serious ^a^	Not serious ^c^	Not serious ^c^	Very serious ^e^	Not assessed ^f^	⊕⊕OO Low
Glutamine	1	Not serious ^a^	Not serious ^c^	Not serious ^c^	Very serious ^e^	Not assessed ^f^	⊕⊕OO Low
**Pre-to-Post i-FABP Outcomes**							
Carbohydrates	4	Not serious ^a^	Very serious ^b^	Not serious ^d^	Very serious ^e^	Serious ^g^	⊕OOO Very low
Probiotics	2	Not serious ^a^	Not serious ^d^	Not serious ^d^	Very serious ^e^	Very Serious ^h^	⊕⊕OO Low
Glutamine	4	Not serious ^a^	Not serious ^d^	Not serious ^d^	Very serious ^e^	Serious ^g^	⊕⊕OO Low
Cystine + Glutamine	1	Not serious ^a^	Not serious ^c^	Not serious ^c^	Very serious ^e^	Not assessed ^f^	⊕⊕OO Low
Bovine colostrum	3	Not serious ^a^	Not serious ^d^	Not serious ^d^	Very serious ^e^	Serious ^g^	⊕⊕OO Low
Curcumin	1	Not serious ^a^	Not serious ^c^	Not serious ^c^	Very serious ^e^	Not assessed ^f^	⊕⊕OO Low
Sodium nitrate	1	Not serious ^a^	Not serious ^c^	Not serious ^c^	Very serious ^e^	Not assessed ^f^	⊕⊕OO Low
High flavonoids	1	Not serious ^a^	Not serious ^c^	Not serious ^c^	Very serious ^e^	Not assessed ^f^	⊕⊕OO Low
Collagen peptides	1	Not serious ^a^	Not serious ^c^	Not serious ^c^	Very serious ^e^	Not assessed ^f^	⊕⊕OO Low
**Exercise Outcomes**							
Time to exhaustion	4	Not serious ^a^	Very serious ^b^	Not serious ^d^	Very serious ^e^	Serious ^g^	⊕OOO Very low
Time trial	3	Not serious ^a^	Serious ^a^	Not serious ^d^	Very serious ^e^	Serious ^g^	⊕⊕OO Low

^a^ Not downgraded, as there was a low risk of bias. ^b^ Downgraded by two levels due to large heterogeneity and CIs. ^c^ Not downgraded, as there is no heterogeneity or indirectness reported, including single-study outcomes. ^d^ Not downgraded due to low heterogeneity and low indirectness (low PICO across studies). ^e^ Downgraded by two levels due to wide CIs and low event rates. ^f^ Not assessed due to only one study included. ^g^ Downgraded by one level due to selective reporting or lack of unpublished results. ^h^ Downgraded by two levels due to selective reporting or lack of unpublished results, asymmetry in funnel plots, or significant industry funding.

## Data Availability

All data are available within the manuscript; specific data queries may be available upon request.
